# Influence analysis of Github repositories

**DOI:** 10.1186/s40064-016-2897-7

**Published:** 2016-08-05

**Authors:** Yan Hu, Jun Zhang, Xiaomei Bai, Shuo Yu, Zhuo Yang

**Affiliations:** School of Software, Dalian University of Technology, Development Zone, Dalian, 116620 China

**Keywords:** Social coding, Github, HITS, Influence analysis

## Abstract

With the support of cloud computing techniques, social coding platforms have changed the style of software development. Github is now the most popular social coding platform and project hosting service. Software developers of various levels keep entering Github, and use Github to save their public and private software projects. The large amounts of software developers and software repositories on Github are posing new challenges to the world of software engineering. This paper tries to tackle one of the important problems: analyzing the importance and influence of Github repositories. We proposed a HITS based influence analysis on graphs that represent the star relationship between Github users and repositories. A weighted version of HITS is applied to the overall star graph, and generates a different set of top influential repositories other than the results from standard version of HITS algorithm. We also conduct the influential analysis on per-month star graph, and study the monthly influence ranking of top repositories.

## Background

The rapid development of social coding tools is leading to a revolution in software product development. Social interactions have become an important factor in the evaluation of the software development process.

Version control systems (VCS) are the essential part of a social coding platform. Nowadays, various VCS tools, e.g. CVS, SVN, Git and etc., are frequently used by software development teams. With them, decentralized team work is possible, and the development process becomes more productive. Software developers can work on their own versions, and submit changes into the decentralized VCS systems. Different versions of software are managed by the VCS system, and potential conflicts of software products are avoided.

Early VCS systems are used only by relatively small software development teams, and are mostly deployed within small area networks, like company LANs. The number of projects maintained within those early VCS systems is also relatively small. As Git can make distributed coding collaboration easier, it is gaining its popularity.

With the recent advances in Internet and cloud computing technology, distributed social coding receives a big boost. Popular social coding platforms can now host millions of software projects. Nowadays, more and more people accept the idea of “social coding”. Contributions to a software development process are most likely made or to be made by a distributed, collaboration-motivated virtual community. Software developers across the world can take part in the same software project, modifying different parts of the code and generating different branches in the project source tree. There are now no explicit boundaries of a software team. A software project may be developed by an ever-changing set of software engineers, and a software engineer may contributed to a set of different software projects hosted in a remote server.

Social coding has tremendously changed the style of software development activities. The social network of software developers continuously interacts with the life cycle of software projects. There have been several social coding platforms that facilitate software engineers around the world to contribute to software projects together. Distributed development tools, e.g. Git, act as the foundation of social coding platforms. Based on Git, the Github platform has attracted many developers to work on millions of open source software projects. In Github, projects have evolved into repositories. Repositories have more information inside. The number of Github users and repositories keep growing.

Github is not only a host of software projects, but also a data source that records software development activities. Many researchers perform analysis on Github Repositories and Github data. Some investigate the collaboration of Github users based on their activities on repositories (Avelino et al. 2015; Jurado and Marín 2015; Lima et al. 2014; Vasilescu et al. 2015b). Some study language importance, or predict the trends of popular programming languages (Casalnuovo et al. 2015; Ray et al. 2014).

As an open social coding platform, there are no restrictions to the creation of new users and repositories. New developers keep coming into Github, new public repositories are being created from time to time. It is now a more important issue to pick out capable or influential ones from millions of Github users. Naturally, the expertise level of a developer is judged by the quality of repositories owned by him, and by his contributions made to Github repositories. Ranking the importance of Github repositories, is thus an necessary work for the evaluation of the Github ecosystem.

In Github, each repository is associated with a set of meta information. The size of the repository, the set of people who starred the repository, etc., are provided by the open Github API. The direct ranking of Github repository based on the size, number of stars, number of forks have been studied. However, ranking of repositories considering social relations in the Github platform, has not been studied yet.

In this paper, we analyzed the importance of Github repositories by considering the social relationship between users and repositories. We consider the two important features of Github Repositories: star, and fork. We use the star relationship to create a star graph, and apply social analysis algorithms on the star graph. The results are then analyzed and the social influence factor of Github repositories are calculated.

The major contributions of this paper include:

1. We built a data acquisition module, which collects Github data from multiple data sources. The retrieved data is processed, and used to build the important social graphs.
2. We proposed a HITS based repository influence analysis, on the star graph constructed from the star relationship between Github users and repositories.
3. We evaluated the weighted version of HITS algorithm. By comparing the results, we found that more reasonable ranking is generated by combining the fork number and the star relationship.
4. We proposed a language-specific analysis, and evaluated the difference of the programming language influence on Github repositories.

## Background

In this paper, we analyze the importance of software repositories using social analysis techniques. In this section, we will present some background information, including link analysis, social coding platform, and the Github timeline data.

### Link analysis algorithms

The basic idea in this paper is to perform social influence analysis on Github repositories using link analysis techniques. Link analysis is first used in ranking web pages. HITS and PageRank are the two major link analysis algorithms, which we will explain in some detail.

#### PageRank

PageRank is a link analysis algorithm used to rank the result pages of Google search engine (Kaplan 2008). PageRank was named after one of the founders of Google, Larry Page.

PageRank is a way of measuring the importance of Web site pages. Google definition: “PageRank works by counting the number and quality of links to determine a rough estimate of how important the web site is. The underly assumption is that more important web sites are likely to receive more link from other web sites”.

The rank of a Page A is described as:

$$\begin{aligned} \hbox {PR(A)} = (1-\hbox {d}) + \hbox {d} (\hbox {PR} (\hbox {T1})/\hbox {C} (\hbox {T1}) + \cdots + \hbox {PR} (\hbox {Tn})/ \hbox {C}(\hbox {Tn})) \end{aligned}$$

The rank of pages are calculated iteratively until the result converges.

#### HITS

Hyperlink-Induced Topic Search (HITS) is a link analysis algorithm which is proposed in 1999, by Dr. Jon Kleinberg of Cornell University (Kleinberg 1999). HITS algorithm divides the Web pages into two types, namely hub pages and authority pages. The authority pages are generally recognized as the important pages on a particular topic. The hub pages, which can be regarded as the pages of evaluating pages, are the pages that link to a collection of authority pages on a particular topic. There is a mutually reinforcing relationship between authority pages and hub pages: a good authority pages should be pointed to by many hub pages, while a good hub page should point to many authority pages. HITS algorithm makes use of the mutually reinforcing relationship between them and gets the page ranks by an iterative computation loop. During the iterative computation, authority weight and hub weight are recalculated and updated, until the values converge.

We adopt HITS algorithm as the basic social analysis technique, and improve HITS algorithm with Github meta information as weights.

### Social coding platform

Distributed coding tools, including CVS, SVN, GIT, have changed the ways of software development. Those social coding platforms have become containers for software collaborations, among software developers on software repositories. Several social coding platforms, including SourceForge and GoogleCode, have contributed to the prosperity of open source projects. As more and more people are used to code maintenance with Git, the Git-backed coding hosting platform now attracts millions of developers to put their software projects there.

Github is a Web based Git repository hosting service, which offers all of the distributed version control and source code management (SCM) functionality of Git. Github provides a Web based graphical interface. It also provides access control and several features such as bug tracking, feature requests, task management, and wikis for every project. Github provides star, fork functionalities to make Github users and repositories form a real social network.

### Github timeline

Although there are other hosts of open source projects that also advocate social coding, like bitbucket and gitorious, Github is still the most popular one.

In Feb 2012, Github publicly announced that its timeline data is available on big query for analysis. Moreover, it offers prizes for the best visualization of the data.

Github provides the social interaction data for free. It faithfully records important actions a Github user performed on repositories. A clean API is provided for interesting people to access the event data. Project timeline can be constructed from those Github events. This functionality makes Github even more popular, not only as a software project hosting service, but also as a target of software engineering research.

## Github data analysis

As the Github platform is becoming popular, analyzing the social activities on Github platform is a new trend in software engineering (Lima et al. 2014). People observe user activities on Github repositories, and analyze the Github repository features to gain insights into the Github data. Two broad categories of research work are closely related to the work in this paper: user collaboration, and repository analysis.

Hauff and Gousios (2015) observe the activities of users on Github, and conduct quantitative analysis of user’s skills and interests based on the observation. Casalnuovo et al. (2015) take a step further, and try to relate the social links between users and users’ language experience to the productivity of developers. User following relationship demonstrates user’s interests to other Github users. Yu et al. (2014) mine from follow networks, and discover several social patterns on Github. People are also interested in other social features of Github users, e.g. leadership, team diversity, gender diversity. McDonald et al. (2014) explore the concepts of distributed leadership, and propose a theory of leadership sharing, to support a model of developer contribution to open source projects. Vasilescu et al. (2015b) present a large data set of social diversity attributes of programmers in Github teams, for researchers to study the effect of team diversity in decentralized teams. Vasilescu et al. (2015a) also study the correlation of gender and tenure diversity to team productivity. Their results show that the gender and diversity are positive predictors of productivity.

As Github repositories are important assets of Github users, their popularity and quality are strong indicators of their owner’s capability. Therefore, analysis of Github repositories becomes one important research branch. Researchers studied variant features of Github repositories, trying to analyze them from different aspects. Jurado and Marín (2015) perform a study over the project issues with Github repositories. They observe the sentimental aspects of Github project issues. Yu et al. (2015) study the pull requests, discuss the complex issue of pull request evaluation latency on Git enabled social coding platforms. Avelino et al. (2015) study the truck factor of popular Github repositories. A project’s truck factor is the number of developer it would need to lose to destroy its progress. Cosentino et al. (2014) evaluates the openness of Github projects with three metrics: the distribution of the project community, the rate of acceptance of external contributions, and the time it takes to become an official collaborator of the project. Tsay et al. (2014) study how to evaluate contributions on Github.

Recent works on Github analysis have revealed many secrets in Github data. However, we found that more efforts should be made to combine social interactions and Github repository features, in order to give a reasonable ranking of Github repositories.

There have been work on evaluating the popularity of Github users (Xavier et al. 2014). We focus on analyzing the popularity (influence) of Github repositories. Similar to the work on evaluating the effect of programming languages on open source projects (Ray et al. 2014), we build language-specific social graph, and conduct language-specific analysis to get the per-language repository influence. People are also interested in the dynamics of Github data. Loyola and Ko (2014) evaluated how the contributor groups on a Github project evolves over time. Considering the evolution nature of Github activities, we also perform an evolutionary study of repository influence ranking.

## Github data collection and social graph construction

What we want to do is to analyze the influence of Github repository based on the public Github timeline data. In the analysis process, we first collect the Github events data that are publicly available through the Github API. Then we extract all the star events and create a star graph to capture the social interactions with regard to user-star-repository actions. Finally, we apply HITS based link analysis on the graph to calculate the influence ranks of repositories.

### Github data collection

The public Github data forms the basis of our analysis. There are now a huge amount of users and repositories on Github, and the number is growing rapidly. Up till now, there are more than 20 million public repositories and millions of users. Those users continuously generate new data upon repositories hosted on Github.

**Fig. 1:**
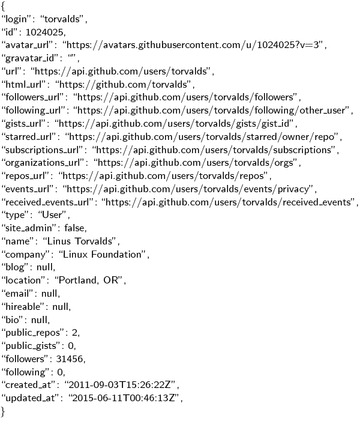
User meta information example. Display the meta information of a Github user by accessing the Github API with url=“https://api.github.com/users/Torvalds”

**Fig. 2:**
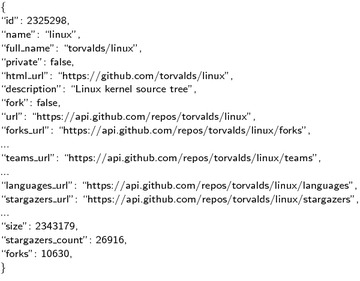
Repo meta information example. Display the meta information of a Github repository by accessing the Github API with url=“https://api.github.com/repos/Torvalds/linux”

In order to analyze the social behavior on Github, the Github data has to be collected first. For each user or repository, Github provides its meta information, like Figs. 1 and 2.

User activities on Github are represented by variant Github events. Github records each user action as an event, and events are generated continuously as time elapses. All the events forms an event stream. There are several kinds of Github events, each representing an important kind of action performed during the software development process. They are:

1. user creation event;
2. repository creation event;
3. commit event;
4. fork event;
5. star event.

Github provides an easy-to-use API to enable access to events data on public repositories. Events data are wrapped in JSON format documents, and can be retrieved by accessing an URL through an HTTP connection.

For practical reasons, Github only makes available the most recent events (events that happened in recent 3 months at most) via the Github API. Therefore, we have to keep crawling the events data every 2 or 3 months. The crawling process is as shown in Algorithm 1. 
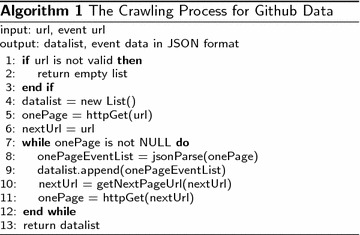


### Github social graph creation

In this paper, the analysis is performed on the star graph. The star graph is constructed from the star relationship between Github user and Github repository. The star graph is created from star events within a given time interval. The creation of star graph is shown in Algorithm 2. 
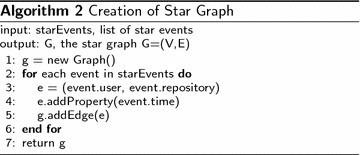


### Social analysis

**Fig. 3:**
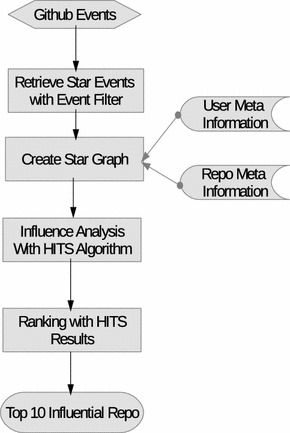
Workflow of the HITS based social analysis. Display the general workflow of the social influence analysis on Github repositories

The main analysis part is performed on the star graph. As the star graph is a bipartite graph with two types of nodes: user and repository, our analysis is based on the HITS algorithm. The whole analysis is illustrated in Fig. 3.

The whole social analysis process is composed of three major steps:

1. data collection. We collect the Github events data, and retrieve star events.
2. create the star graph. The star graph represent the social structure that our social influence analysis is based upon.
3. HITS based social analysis. The details of the social analysis algorithms will be discussed in the next section.

## HITS based repository influence analysis

In this section, we present the HITS based social influence analysis for Github repositories. The details of the HITS based analysis are presented. Apart from the basic HITS analysis, we also discuss how to perform language-specific analysis, and how to use the Github meta information to build weighted HITS analysis of Github repositories.

### HITS based analysis

The basic form of HITS algorithm consists of two main processes: constructing the adjacency graph and computing the authority weights and hub weights iteratively. We use an undirected graph G = (V, E), to represent the star graph which shows the star relationship between Github users and repositories. The nodes of the graph is of two types, we make it V = V(u) + V(r). V(u) stands for Github users who have starred some Github repositories. V(r) stands for Github repositories that have been starred at least once. An edge e = (user, repo) belongs to E, meaning user stars repo at certain time.

**Fig. 4:**
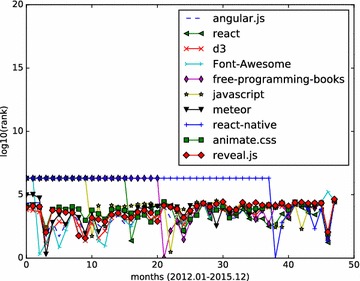
Monthly rank curve of top 10 HITS repositories. Display the monthly ranking dynamics of the top 10 repositories returned by HITS algorithm

For each node v, two weights are assigned to it: a(v) means the authority weight, and h(v) means the hub weight. In each computation step, the two weight values of a node is updated following two rules:

1. a(v) = sigma h(v)
2. h(v) = sigma a(v)

The basic HITS algorithm implemented is as Algorithm 3: 
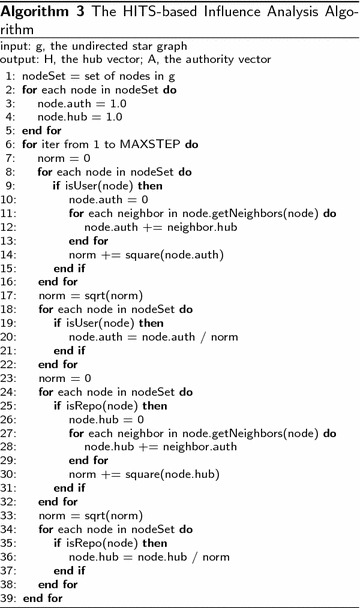


To perform topic distillation, we create language specific star graph for major languages. We then apply HITS algorithm on those language-specific graphs to analyze the influence of repositories in different programming languages.

In order to perform language-specific analysis, we will have to first retrieve the meta information of Github users and repositories. A Github user has many properties, which can be accessed via Github API with URL of special format. In this paper, we would like to retrieve the meta information and store it in a database, which will be used later in influence analyses. We wrap the interesting features in a tuple t = (uid, uname, enterDate). The steps of getting this tuple is shown in Algorithm 4. 
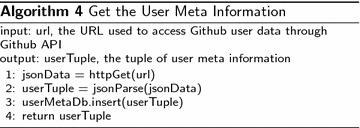


The database userMetaDb will later be used to attract user id, and user names.

Similarly, the repository meta information can also be retrieved through the Github API. Given a URL of a Github repository, we would get the meta information of the repository with the process described in Algorithm 5. 
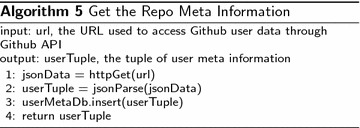


The process of creating language-specific star graph is defined in Algorithm 6. 
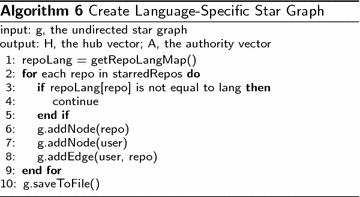


### Weighted HITS analysis

For Github ecosystem, treating all the link information of user-star-repository relationship as the same may not be appropriate. The importance of Github repositories vary. One important factor is the fork counts and size of Github repositories.

We can use the features of Github repository as weights and perform weighted HITS analysis. Line 26 of Algorithm 3 should change to “node.hub = w”, where the node’s hub value is initialized with the weight w.

### Improve HITS algorithm with repository’s fork information

The forking rate of Github repository is deemed as one of the most important features to indicate the popularity of a specific fork count.

The fork information comes from the Github event data. Algorithm 7 shows how the repository fork information is built from Github event data. 
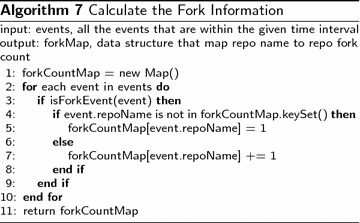


## Experiments and evaluation

Experimental setup for the Github social influence analysis is presented in this section. Firstly, The setup of data collector and experimental environments are explained in detail. Secondly, we present the results of basic HITS analysis and weighted analysis on the complete star graph. Thirdly, we present the monthly influence analysis of Github repositories, and track the dynamics of repository ranking.

### Dataset setup

We collect the experimental data from two data sources: the original Github API, and githubarchive Web site. The meta information about Github users and repositories are retrieved with the Github API. This work is done by scraping the Github data URLs.

We get the Github events data from githubarchive. The timeline data keeps growing, and needs to be crawled every 2–3 months due to Github restrictions. As Github oriented analysis is becoming popular, there are special archive site focusing on crawling Github data continuously and archive the data for researchers to download. Ghtorret and githubarchive are the two typical Github data archive site, which have been used in recent studies on Github analysis.

### Experimental setup

The experiments are conducted on Intel i5, BSD Unix, 8G RAM. The star graph is created using the SNAP library. Currently, we construct the star graph with 4 years star events data from Jan 1st, 2012 until Dec 31, 2015. SNAP is a C++ library that facilitates social analysis on large graphs. We use the Python wrapper for SNAP, and connects with python Github database.

### Analysis of Github repositories

The top 10 Github Repositories returned by the standard HITS algorithm implementation in SNAP library are listed in Table 1. Most of these top 10 repositories are JavaScript or HTML5 web applications. It shows that considering only star relationship during the evaluated time interval, JavaScript based web applications are overwhelmingly more popular than repositories implemented with other programming languages.

**Table 1:** Top 10 Github repositories with HITS algorithm

Rank	Repo name	Hub value
1	angular/angular.js	0.0447933608671
2	facebook/react	0.0429770574216
3	mbostock/d3	0.0411178537051
4	FortAwesome/Font-Awesome	0.0399144170895
5	vhf/free-programming-books	0.0397495680716
6	airbnb/javascript	0.0358427149496
7	meteor/meteor	0.0334891188394
8	facebook/react-native	0.0311704638485
9	daneden/animate.css	0.031147531885
10	hakimel/reveal.js	0.03028518744

When we apply the fork-weighted HITS algorithm on the 4-year star graph, we get different top repositories, as listed in Table 2. When the fork factor is considered together with the star relationship, highly forked repositories with reasonable amounts of stars get in the top 10 list. The repository “octocat/Spoon-Knife” is not an actual feature rich repository, it is a repository used as a demo of the Github “fork” feature. It has a very high fork rate, it is why it appears as one of the top repositories in the results. Highly forked online course projects like “rdpeng/ProgrammingAssignment2”, “DataScienceSpecialization/courses”, show in the top 10 fork-weighted list. The results shows that they have high social influence, although they are not hot software development projects.

**Table 2:** Top 10 Github repositories with fork-weighted HITS algorithm

Rank	Repo name	Hub value
1	jtleek/datasharing	0.664059586365
2	rdpeng/ProgrammingAssignment2	0.393534756
3	octocat/Spoon-Knife	0.365773402631
4	twbs/bootstrap	0.186108421832
5	rdpeng/ExData_Plotting1	0.170712175547
6	rdpeng/RepData_PeerAssessment1	0.104721087113
7	angular/angular.js	0.10327750696
8	DataScienceSpecialization/courses	0.0956450957915
9	udacity/frontend-nanodegree-resume	0.0697668647334
10	Homebrew/homebrew	0.0662650437365

We also tried size-weighted HITS algorithm on the 4-year star graph, and the results are shown in Table 3. It seems that the size of a repository alone is not good indicator for repository influence. Well-known large-scale influential repositories, like “cdnjs/cdnjs”, “WebKit/webkit” do not appear in the top 10 list.

**Table 3:** Top 10 Github repositories with size-weighted HITS algorithm

Rank	Repo name	Hub value
1	alphaHeavy/bloomberg_symbols	0.033543664973
2	jj1bdx/tinymtdc-longbatch	0.031942377449
3	practicalswift/osx	0.03161613091
4	Tmustafaramadhan/kloxo	0.029476151813
5	MyCATApache/Mycat-download	0.0287408749217
6	mkalin/jwsur2	0.0283066388087
7	MiCode/patchrom_miui	0.0267653188291
8	angular/angular.js	0.0265660417786
9	kiang/bulletin.cec.gov.tw	0.0264688182206
10	JustArchi/ArchiDroid-legacy	0.0261632821478

### Monthly analysis of Github repository ranking

Popular Github repositories keep attracting attentions of capable software developers. The influence of a repository may change overtime. Based on this observation, we analyze the Github events data in a monthly fashion.

**Fig. 5:**
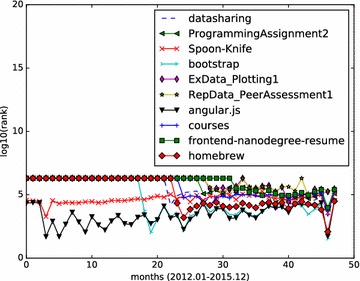
Monthly rank curve of top 10 fork-weighted HITS repositories. Display the monthly ranking dynamics of the top 10 repositories returned by the fork-weighted HITS algorithm

**Fig. 6:**
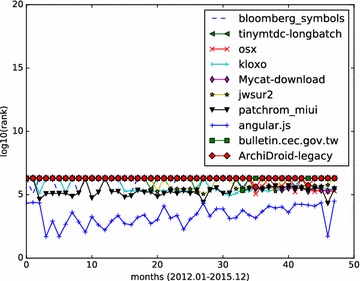
Monthly rank curve of top 10 size-weighted HITS repositories. Display the monthly ranking dynamics of the top 10 repositories returned by the size-weighted HITS algorithm

Monthly star graphs are built from the monthly events data. HITS algorithm is applied to those star graphs. We focus on the top influential Github repositories, and demonstrate how a repository’s influence value varies month by month. We calculate the hub values of all starred repositories, and rank those repositories according to their hub values. The analysis results (monthly ranking curve) on the three set of top repositories are illustrated in Figs. 4, 5 and 6.

In Fig. 4, we study the top 10 ranked repositories generated with the standard HITS algorithm implemented within the SNAP library. The results show the necessity of analyzing the Github timeline data month by month.

1. angular/angular.js is a very popular HTML client-side enhancement library. It keeps a relatively stable high rank during the observed months.
2. facebook/react: A declarative, efficient, and flexible JavaScript library for building user interfaces. It is another JavaScript project with steady rank lines.
3. hmbostock/d3: It achieves relatively high ranks in all the 48 months.
4. FortAwesome/Font-Awesome: It is a popular font and css toolkit.
5. vhf/free-programming-books: It is an authoritative page that keeps freely available programming books. It is popular information source, but not a software project. It gets its first top1 rank in month 21, and after then stays popular as the monthly ranking indicates.
6. airbnb/javascript: It is a JavaScript style guide.
7. meteor/meteor: It is a JavaScript App platform. It keeps high influential rank during 2012, then steady relatively high rank till Dec 2015.
8. facebook/react-native: It is a framework for building native apps with React. It is a newly popular application March 2015.
9. daneden/animate.css: It is a cross-browser library of CSS animations. It keeps relatively steady high rankings throughout the 4 years.
10. hakimel/reveal.js: It is a popular HTML representation framework. It keeps relatively steady high rankings throughout the 4 years.

Figure 5 shows the monthly ranking of the fork-weighted HITS top 10 results. Those 10 repositories presents steady ranks than those in Fig. 4. The repository octocat/Spoon-Knife is a high-forked repository, but is not actually popular. It is a project with few files, and has a small size. As we check it out, it is a project that is made typically as an example to demonstrate the fork feature of Github. This explains why it has high forks while exhibiting low monthly influence ranks. All other applications comes with monthly ranks that fit well with the fork-weighted ranks.

We also studied the monthly ranking of the top 10 size-weighted HITS algorithm results. Figure 6 shows the monthly ranking of the size-weighted HITS top 10 results. As shown in Fig. 6, Github repositories of very large size tend to be starred by fewer developers. Among the top 10 repositories returned by size-weighted HITS algorithm, only three repositories (practicalswift/osx, Tmustafaramadhan/kloxo, angular/angular.js) are continuously influential during the 4 years.

From the results depicted by Figs. 4, 5, 6, we can see that the social influence of a repository tends to change month by month. For those top influential repositories, they won’t be high influential each month. However, they often have a steady influential rank, while having high ranks in several months.

### Language-specific monthly analysis of Github repository ranking

The monthly HITS based influence analysis is also applied to repositories of several major programming languages, including JavaScript, Python, Java, PHP.

We retrieved the top 10 results with HITS algorithm and fork-weighted algorithm and perform monthly analysis for top JavaScript and Python repositories. Monthly rank values of language-specific repositories are calculated according to their hub values.

We first build a star graph for JavaScript repositories, and perform HITS analysis on it. The full names of top 10 influential JavaScript repositories return by HITS algorithm are:

1. angular/angular.js;
2. facebook/react;
3. mbostock/d3;
4. airbnb/javascript;
5. meteor/meteor;
6. hakimel/reveal.js;
7. adobe/brackets;
8. getify/You-Dont-Know-JS;
9. driftyco/ionic;
10. bartaz/impress.js.

The full names of the top 10 influential JavaScript repositories returned by the fork-weighted HITS algorithm are:

1. twbs/bootstrap;
2. angular/angular.js;
3. DataScienceSpecialization/courses;
4. udacity/frontend-nanodegree-resume;
5. gabrielecirculli/2048;
6. mbostock/d3;
7. heroku/node-js-sample;
8. jquery/jquery;
9. tastejs/todomvc;
10. h5bp/html5-boilerplate.

**Fig. 7:**
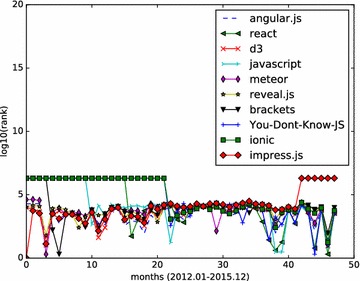
Monthly rank curve of top 10 HITS repositories (JavaScript). Display the monthly ranking dynamics of the top 10 JavaScript repositories returned by the HITS algorithm

**Fig. 8:**
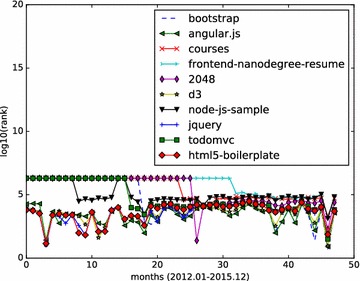
Monthly rank curve of top 10 fork-weighted HITS repositories (JavaScript). Display the monthly ranking dynamics of the top 10 JavaScript repositories returned by the fork-weighted HITS algorithm

The JavaScript repositories ranking in months are illustrated in Figs. 7 and 8.

Two popular repository, angular/angular.js, stand out top in the results of standard HITS and fork-weighted HITS algorithms.

The fork-weighted monthly results in Fig. 8 shows that highly-forked JavaScript repositories also receives increased stars.

For Github repositories implemented with Python, a similar language-specific analysis is conducted. The full names of top 10 Python repositories returned by the HITS algorithm are:

1. vinta/awsome-python;
2. kennethreitz/requests;
3. mitsuhiko/flask;
4. django/django;
5. ansible/ansible;
6. scrapy/scrapy;
7. rg3/youtube-dl;
8. rethinkdb/rethinkdb;
9. faif/python-patterns;
10. mrdoob/three.js.

The full names of top 10 influential Python repositories returned by the fork-weighted HITS algorithm are:

1. shadowsocks/shadowsocks;
2. mrdoob/three.js;
3. django/django;
4. bitcoin/bitcoin;
5. mitsuhiko/flask;
6. scikit-learn/scikit-learn;
7. ansible/ansible;
8. julycoding/The-Art-Of-Programming-By-July;
9. numbbbbb/the-swift-programming-language-in-chinese;
10. scrapy/scrapy.

**Fig. 9:**
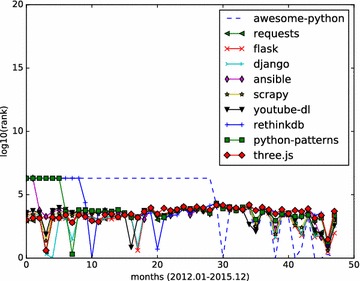
Monthly rank curve of top 10 HITS repositories (Python). Display the monthly ranking dynamics of the top 10 Python repositories returned by the HITS algorithm

**Fig. 10:**
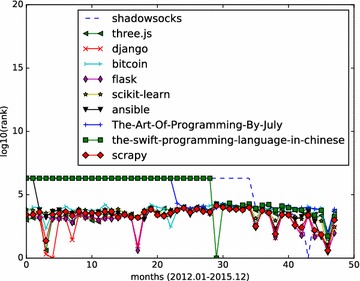
Monthly rank curve of top 10 fork-weighted HITS repositories (Python). Display the monthly ranking dynamics of the top 10 Python repositories returned by the fork-weighted HITS algorithm

The Python repositories ranking in months are illustrated in Figs. 9 and 10. The results show that the top 10 results of fork-weighted HITS algorithm give more reasonable ranks to popular Python repositories on Github. Projects like django/django, scikit-learn/scikit-learn, scrapy/scrapy are in the top ranks, and they are real popular projects that are well known to python programmers.

## Conclusion

Github has become the most popular social coding platform. As such, it is an interesting issue to analyze the social factors of Github users and repositories. In this paper, we present HITS based influence analysis on Github repositories. We design Github data collection module, which collects github data from multiple data sources. Our data collector retrieves the Github events from githubarchive.org. We then filter out star events from the collected event stream, and build the star graph to capture the basic social relationship between Github users and repositories. Meta information about Github users and repositories, are crawled from Github API urls, also by our data collector. Important attributes are retrieved from those meta information and used in fork-weighted and size-weighted HITS algorithms proposed in this paper. We evaluated those HITS based algorithms on the star graphs generated from Github data. In order to demonstrate the dynamics of repository influence, we design per-month HITS analysis and draw curves of monthly repository ranking. And we also create language-specific star graphs, and perform HITS ranking and monthly ranking evaluation for popular languages like JavaScript and Python. The findings show that the adapted HITS algorithm and monthly analysis gives more insights into the social influence of Github repositories. Based on the results, we can develop new analyses, like detection of patterns or trends of Github social influence, detection of anomalies in Github social activities, classification of Github repositories.
